# Global *Shigella* epidemiology and vaccine coverage stratified by socioeconomic development levels, 1934–2025

**DOI:** 10.1128/spectrum.02393-25

**Published:** 2026-08-04

**Authors:** Jiani Jiang, Yi Yang, Xiangkun Zeng, Chen Xu, Xiaofan Li, Yuanyuan Li, Yunbing Li, Ning Dong

**Affiliations:** 1 Department of Emergency Medicine, Second Affiliated Hospital, Zhejiang University School of Medicine 26441, Hangzhou, China; 2 Department of Medical Microbiology, School of Basic Medical Sciences, Suzhou Medical College of Soochow University 74565, Suzhou, China; 3 Department of Laboratory Medicine, School of Medicine, Jiangsu University 191611, Zhenjiang, China; 4 Department of Medical Microbiology, Experimental Center, Suzhou Medical College of Soochow University 74565, Suzhou, China; University of Hong Kong, Hong Kong, Hong Kong

**Keywords:** antimicrobial resistance, molecular epidemiology, *Shigella*, socioeconomic development

## LETTER

The prominence of *Shigella* was underscored by the 2024 WHO Bacterial Priority Pathogens List (BPPL), which elevated fluoroquinolone-resistant *Shigella* spp. to the high-priority group due to its burden and increasing resistance in low- and middle-income countries (LMICs) (1). In economically transitioning countries, *Shigella* dominance has shifted between *S. flexneri* and *S. sonnei*, emphasizing the need for further investigation of *Shigella*’s global epidemiology and its correlation with socioeconomic factors (2).

We retrieved all available *Shigella* genome sequences from the NCBI GenBank database (*n* = 49,228; 29 April 2025). After filtering for complete temporal and geographic data, 43,180 isolates, collected between 1934 and 2025 in 70 countries across 6 continents, were retained (Table S2). Over the past century, *Shigella* has undergone substantial changes in species distribution, serotype diversity, antibiotic resistance, and the carriage of the virulence plasmid pINV (3). A significant epidemiological shift occurred between 2014–2019 and 2020–2024 (*χ*^2^ = 1,472.3, *P* < 0.0001). *S. flexneri* replaced *S. sonnei* as the dominant strain, peaking at 67.06% in 2021. Conversely, *S. boydii* and *S. dysenteriae* remained rare. The prevalence of *S. flexneri* 1b increased significantly from 0.05% in 2013 to 12.13% in 2024, while *S. flexneri* 2a remained stable at 15%–25%. The pINV carriage rate increased steadily from 35.58% in 2014 to 55.69% in 2019, followed by a sharp surge to 69.44% in 2021, remaining at 69%–70% through 2024.

A total of 214 antimicrobial resistance genes spanning nine classes (aminoglycosides, polymyxins, quinolones, β-lactamases, chloramphenicol, sulfonamides, macrolides, fosfomycin, and tetracyclines) were identified. *bla_CTX-M-15_* demonstrated sustained growth, increasing from 8.74% in 2014 to 16.28% in 2021. *bla*_CTX-M-14_ showed pronounced fluctuations, peaking at 4.23% in 2018 before returning to baseline levels. *bla_CTX-M-55_* and *bla_CTX-M-3_* remained at low prevalence levels. Fluoroquinolones and macrolides, the WHO-recommended therapeutics for shigellosis, require ongoing surveillance of their resistance gene carriage. The *qnr* demonstrated a general increase in prevalence, notwithstanding a transient decline to 9.96% in 2019, before peaking at 24.35% in 2020. The prevalence of *mph*(A) fluctuated between 23.93% and 32.84% from 2014 to 2024, while *erm*(B) steadily declined from 22.45% in 2017 to 21.17% in 2024. Additionally, the mobile colistin resistance gene *mcr-1* transitioned from exclusive detection in *S. flexneri* to predominant occurrence in *S. sonnei* after 2021 (Table S1).

The Human Development Index (HDI) classifies countries into four levels: very high (VHHD), high (HHD), medium (MHD), and low human development (LHD) (4). In VHHD and HHD regions, *S. sonnei* predominated (51.16% and 64.90%), followed by *S. flexneri* (46.01% and 33.01%). Conversely, *S. flexneri* was dominant in MHD and LHD regions (63.21% and 68.07%), significantly outnumbering *S. sonnei* (23.63% and 22.89%). *S. boydii* and *S. dysenteriae* were more prevalent in MHD (9.81% and 3.34%) and LHD (6.93% and 2.11%) areas. Serotype 2a was most common, with serotype 6 showing higher prevalence in MHD (8.14%) and LHD (10.84%) areas. Based on this serotype distribution, we estimated the potential coverage of leading *Shigella* vaccine candidates in each HDI stratum. Due to the antigenically uniform *S. sonnei* in VHHD and HHD regions, the theoretical coverage ranged from 69.70% to 84.81%. In contrast, coverage dropped to 41.14%–64.16% in MHD and LHD regions, primarily due to the high burden and serotypic diversity of *S. flexneri*. These findings highlight the importance of region-specific vaccine strategies to ensure broad and equitable protection (Table 1) (3).

**TABLE 1:** Distribution of *Shigella* species and vaccine coverage in regions with different levels of human development index

*Shigella* species and vaccine coverage	Regions with different human development index				*P* value^*b*^
	Very high human development region (*n* = 38,102)^*a*^	High human development region (*n* = 2,345)	Medium human development region (*n* = 897)	Low human development region (*n* = 332)	
Species and serotypes					
*S. flexneri*	17,532 (46.01%)	774 (33.01%)	567 (63.21%)	226 (68.07%)	<0.0001
1a	163 (0.43%)	74 (3.16%)	3 (0.33%)	0 (0.00%)	<0.0001
1b	3,000 (7.87%)	5 (0.21%)	48 (5.35%)	32 (9.64%)	<0.0001
1c (7a)	1,628 (4.27%)	0 (0.00%)	26 (2.90%)	4 (1.20%)	<0.0001
1d	0 (0.00%)	3 (0.13%)	1 (0.11%)	0 (0.00%)	0.001
2a	7,063 (18.54%)	395 (16.84%)	157 (17.50%)	80 (24.10%)	0.022
2b	387 (1.02%)	49 (2.09%)	113 (12.60%)	7 (2.11%)	<0.0001
3a	2,759 (7.24%)	10 (0.43%)	52 (5.80%)	21 (6.33%)	<0.0001
3b	466 (1.22%)	1 (0.04%)	2 (0.22%)	1 (0.30%)	<0.0001
4a	22 (0.06%)	0 (0.00%)	0 (0.00%)	0 (0.00%)	0.467
4av	322 (0.85%)	0 (0.00%)	25 (2.79%)	14 (4.22%)	<0.0001
4b	29 (0.08%)	0 (0.00%)	0 (0.00%)	0 (0.00%)	0.436
4bv	18 (0.05%)	0 (0.00%)	0 (0.00%)	0 (0.00%)	0.569
5a	26 (0.07%)	0 (0.00%)	1 (0.11%)	0 (0.00%)	0.539
5b	35 (0.09%)	9 (0.38%)	38 (4.24%)	0 (0.00%)	<0.0001
6	383 (1.01%)	0 (0.00%)	73 (8.14%)	36 (10.84%)	<0.0001
7b	22 (0.06%)	1 (0.04%)	0 (0.00%)	0 (0.00%)	0.906
X	21 (0.06%)	25 (1.07%)	0 (0.00%)	0 (0.00%)	<0.0001
Xv(4c)	99 (0.26%)	119 (5.07%)	4 (0.45%)	10 (3.01%)	<0.0001
Y	453 (1.19%)	15 (0.64%)	3 (0.33%)	1 (0.30%)	0.008
Yv	20 (0.05%)	13 (0.55%)	0 (0.00%)	0 (0.00%)	<0.0001
Unknown	586 (1.54%)	21 (0.90%)	2 (0.22%)	6 (1.81%)	0.002
*S. sonnei*	19,494 (51.16%)	1522 (64.90%)	212 (23.63%)	76 (22.89%)	<0.0001
*S. boydii*	836 (2.19%)	49 (2.09%)	88 (9.81%)	23 (6.93%)	<0.0001
*S. dysenteriae*	240 (0.63%)	0 (0.00%)	30 (3.34%)	7 (2.11%)	<0.0001
Combination of serotypes in leading vaccine candidates					
*S. flexneri* 2a, 3a, or 6, or *S. sonnei*	29,699 (77.95%)	1,927 (82.17%)	494 (55.07%)	213 (64.16%)	<0.0001
*S. flexneri* 1b, 2a, or 3a, or *S. sonnei*	32,316 (84.81%)	1,932 (82.39%)	469 (52.29%)	209 (62.95%)	<0.0001
*S. flexneri* 2a or *S. sonnei*	26,557 (69.70%)	1,917 (81.75%)	369 (41.14%)	156 (46.99%)	<0.0001

^*a*^ *"n*" is the number of *Shigella* genome sequences downloaded from the NCBI database for each region. Due to the absence of location information for some strains, a total of 41,676 genome sequences were included in the final analysis.

^*b*^ The Chi-square test was used to determine whether each type of bacterial strain showed a statistically significant difference between very high human development, high human development, medium human development, and low human development regions. *P* < 0.05 was considered statistically significant.

Several limitations should be considered. First, inherent to the utilization of NCBI genomic data are geographic and socioeconomic biases, with overrepresentation from high-income countries, affecting the representativeness of global estimates and complicating HDI-based comparisons, making it challenging to fully capture the true *Shigella* burden in low-resource settings. Second, temporal trends in specific resistance genes should be interpreted cautiously. These apparent peaks likely reflect historical sequencing biases and targeted multidrug-resistant surveillance, rather than true epidemiological changes. Additionally, the number of isolates is uneven across decades, characterized by scarce early data and a dramatic post-2000 surge, which potentially biases long-term trend interpretations. Finally, while *Shigella* epidemiology associates with HDI, this ecological shift is multifactorial. Diverse national antibiotic consumption policies and globalization factors, such as international travel, also profoundly impact pathogen prevalence and the cross-border transmission of resistant strains.

In conclusion, our study uncovered an association between *Shigella* epidemiology and socioeconomic development, with *S. sonnei* predominating in high-income regions and S. *flexneri* in LMICs. In the past decade, the dominant *Shigella* species has shifted from *S. sonnei* to *S. flexneri*, accompanied by increasing antibiotic resistance and pINV prevalence. These findings underscore the imperative for tailored, multifaceted *Shigella* control strategies integrating sanitation, healthcare access, and equitable vaccine deployment (5).

## SUPPLEMENTAL MATERIAL

Tables S1 to S3 spectrum.02393-25-s0001.xlsx Table S1: Global characteristics of *Shigella* from 1934-2025. Table S2: Genomic metadata of *Shigella* isolates from 1934 to 2025. Table S3: Temporal distribution of *Shigella* isolates by decade.

ASM does not own the copyrights to Supplemental Material that may be linked to, or accessed through, an article. The authors have granted ASM a non-exclusive, world-wide license to publish the Supplemental Material files. Please contact the corresponding author directly for reuse.
